# Exploring the Nutritional Value and Bioactive Potential of *Craterellus cornucopioides* (L.) Pers. as a Functional Food Source

**DOI:** 10.3390/foods14234124

**Published:** 2025-12-01

**Authors:** Mariana-Gabriela Bumbu, Mihaela Niculae, Irina Ielciu, Adela Pintea, Zsolt Matyas, Ștefan Alexandru Anton, Daniela Benedec, Melinda Fogarasi, Ioan Marcus, Oana Roșca-Casian, Nicodim Fiț, Daniela Hanganu

**Affiliations:** 1 Department of Physiopathology, Faculty of Veterinary Medicine, University of Agricultural Sciences and Veterinary Medicine Cluj-Napoca, Calea Mănăştur 3-5, 400372 Cluj-Napoca, Romania; mariana-gabriela.bumbu@usamvcluj.ro (M.-G.B.); ioan.marcus@usamvcluj.ro (I.M.); 2 Department of Infectious Diseases, Faculty of Veterinary Medicine, University of Agricultural Sciences and Veterinary Medicine Cluj-Napoca, Calea Mănăştur 3-5, 400372 Cluj-Napoca, Romania; 3 Department of Pharmaceutical Botany, Faculty of Pharmacy, “Iuliu Hațieganu” University of Medicine and Pharmacy, 23 Gheorghe Marinescu Street, 400337 Cluj-Napoca, Romania; irina.ielciu@umfcluj.ro; 4 Department of Biochemistry, Faculty of Veterinary Medicine, University of Agricultural Sciences and Veterinary Medicine Cluj-Napoca, Calea Mănăştur 3-5, 400372 Cluj-Napoca, Romania; apintea@usamvcluj.ro (A.P.); zsolt.matyas@student.usamvcluj.ro (Z.M.); stefan-alexandru.anton@student.usamvcluj.ro (Ș.A.A.); 5 Department of Pharmacognosy, Faculty of Pharmacy, “Iuliu Hațieganu” University of Medicine and Pharmacy, 12 Ion Creangă Street, 400010 Cluj-Napoca, Romania; dbenedec@umfcluj.ro (D.B.); dhanganu@umfcluj.ro (D.H.); 6 Faculty of Food Science and Technology, University of Agricultural Sciences and Veterinary Medicine Cluj-Napoca, Calea Mănăştur 3-5, 400372 Cluj-Napoca, Romania; melinda.fogarasi@usamvcluj.ro; 7 Alexandru-Borza Botanical Garden, Babes-Bolyai University, 42 Republicii Street, 400015 Cluj-Napoca, Romania; oana.rosca@ubbcluj.ro; 8 Department of Microbiology, Faculty of Veterinary Medicine, University of Agricultural Sciences and Veterinary Medicine Cluj-Napoca, Calea Mănăştur 3-5, 400372 Cluj-Napoca, Romania; nfit@usamvcluj.ro

**Keywords:** *Craterellus cornucopioides* (L.) Pers., edible fungi, nutritional composition, bioactive compounds, antioxidant properties, antimicrobial efficacy

## Abstract

This study aimed to explore the phytochemical variability, nutrient composition, and bioactive profile of the edible mushroom species *Craterellus cornucopioides* with specimens originating from Romanian flora. Its nutritional profile, including its proximate composition and energy value, was determined using standardized analytical methods. The mean contents of total polyphenols and caffeic acid derivatives, quantified by spectrophotometric assay, were established. HPLC–DAD–ESI^+^ analysis enabled the identification and quantification of individual phenolic constituents. Its antioxidant potential was systematically assessed using the following in vitro complementary assays: FRAP, ABTS, CUPRAC, DPPH, and ORAC. Antimicrobial activity was evaluated in vitro against MSSA, MRSA, *Bacillus cereus*, *Enterococcus faecalis*, *Listeria monocytogenes*, *Escherichia coli*, *Pseudomonas aeruginosa*, and *Candida albicans* using well diffusion, broth microdilution, and anti-biofilm assays. The high amounts of polyphenols, including gentisic acid and protocatechuic acid, underlined a biologically relevant phytochemical composition. In fact, all tested extracts and, in particular, CE3 extract consistently displayed strong antioxidant properties, as indicated by five complementary in vitro tests (FRAP, ABTS, CUPRAC, DPPH, and ORAC). In addition, CE1-4 extracts expressed in vitro antimicrobial potential towards all tested organisms except for *Pseudomonas aeruginosa.* Our results underscore *C. cornucopioides*’ nutritional, antioxidant, and antimicrobial potential, thus supporting its classification as an edible but under-explored mushroom species with promising applications in both the food and pharmaceutical industries.

## 1. Introduction

Nature-based therapeutic approaches are often favored because they tend to have fewer side effects compared to conventional treatments. Traditional Chinese medicine has acknowledged the medicinal potential of mushrooms for centuries, incorporating them into various treatments. Over the years, numerous studies have supported the effectiveness of mushrooms in this regard, reinforcing their role in both traditional and modern medical practices [1]. Nutraceuticals are food components that provide health benefits, including disease prevention and treatment. Mushrooms, prized for their taste and nutritional value, are classified as both functional and nutraceutical ingredients. They are highly valued for their flavor, medicinal properties, and economic importance. The nutritional benefits of edible mushrooms come from their high protein, fiber, vitamin, and mineral content, along with their low fat levels. Additionally, they contain various bioactive compounds that provide medical benefits on multiple levels [2].

Among the edible mushroom species, the species *Craterellus cornucopioides* (L.) Pers., known as the black trumpet, is an edible mushroom belonging to the Cantharellaceae family, commonly found in deciduous forests on acidic soils. It is rich in bioactive compounds, including phenolic acids (gallic, caffeic, rosmarinic), flavonoids (quercetin, myricetin, resveratrol), polysaccharides, terpenoids (craterellins, illudins), sterols (ergosterol), vitamins (B12, C, A, D_3_, E), essential amino acids, unsaturated fatty acids, and minerals (K, Mg, Fe, Zn). These compounds underpin its multiple biological activities, including antioxidant, anti-inflammatory, antimicrobial, antimutagenic, cytotoxic, and immunomodulatory effects [3]. Its polysaccharides activate macrophages via the TLR4–NF-κB pathway, while phenolics contribute to strong radical-scavenging activity. Aqueous extracts also exhibit ACE-inhibitory potential, suggesting antihypertensive benefits. Overall, *C. cornucopioides* represents a nutrient-dense functional food and a promising source of natural therapeutic agents with applications in antimicrobial, anti-inflammatory, and anticancer strategies [4].

The antioxidant properties of mushrooms are primarily attributed to their bioactive compounds, which include an array of phenolic compounds, flavonoids, polysaccharides, vitamins, and minerals. These compounds work synergistically to neutralize free radicals, thereby protecting cells from oxidative stress. Oxidative stress can cause oxidative damage to essential biological molecules such as DNA, proteins, and lipids, and it is linked to numerous chronic diseases, including cancer, cardiovascular diseases, and neurodegenerative disorders [5,6].

The development of antibiotics over the past seventy years has been a major scientific achievement. These compounds work by interfering with various bacterial processes and structures, such as cell wall synthesis, plasma membrane permeability, DNA replication, and protein synthesis. The bacterial cell wall, which provides shape and rigidity and acts as an osmotic barrier, contains varying peptidoglycan levels, about 10% in Gram-negative bacteria and up to 60% in Gram-positive bacteria. Despite the wide range of antibacterial compounds, bacterial resistance to primary antibiotics has significantly increased [6]. The antibacterial activity of mushrooms has been tested and confirmed as an important bioactivity due to their metabolites, isolated from their fruiting bodies, mycelia, or spores [7,8].

The antioxidant activity of *C. cornucopioides* has been previously investigated, especially through in vitro assays, and has been correlated to compounds such as polysaccharides [9,10,11,12,13], organic acids [5], and phenolic compounds [4,14,15,16,17,18]. Additionally, the antibacterial activity of the species has been tested in several studies [8,16,19]. Further investigations are warranted to comprehensively elucidate the polyphenolic composition of *C. cornucopioides* and to expand the current understanding of its biological activities, particularly those related to its antioxidant and antimicrobial potential. In this context, the present study aims to explore the phytochemical variability, nutritional composition, and bioactive profile of *C. cornucopioides* originating from Romanian flora, thereby providing new insights into the species’ functional and nutraceutical value. Specifically, the bioactivities that were explored were its antioxidant and antimicrobial properties. Taking all this into consideration, it appears necessary to deepen our knowledge of the antioxidant and antimicrobial activity of this species. The novelty of the present study lies in the fact that it aims to offer scientific evidence to confirm the existing proof of the bioactivities of this edible mushroom.

## 2. Materials and Methods

### 2.1. Mushroom Sample Collection and Preparation of Extracts

The plant material consisted of mushroom samples collected from three distinct counties of Romania in different years: Cluj, 2022 (CE1); Sălaj, 2022 (CE2); Suceava, 2022 (CE3) and 2023 (CE4). They were harvested in August of each year, with approximately 400 g of fresh mushroom material collected per location. The collected mushrooms were sorted on a clean surface, removing those that were too old, heavily infested with insects, moldy, or decaying, keeping only those that were fresh and healthy. Soil, leaves, and other impurities were removed with a soft brush, and the dirty base of the stem, as well as damaged or insect-infested portions, were cut away with a scalpel or small knife. If insects were found inside, the mushrooms were cut lengthwise and cleaned by hand, then dried on metal racks. These mushrooms were subsequently preserved in a dried form for a few days to maintain their integrity and facilitate further analysis. The plant material was authenticated and identified by Oana Roșca-Casian, PhD, and voucher specimens are preserved at the “Alexandru Borza” Botanical Garden in Cluj-Napoca, Northwestern Romania (voucher number CL 672126). 

The mushrooms were dried for 6 h at 52 °C using an electric food dehydrator (Excalibur 93926TVDB, Sacramento, CA, USA). The process of grinding the dried mushrooms began by thoroughly checking that they were completely dehydrated, ensuring that they were brittle and free of residual moisture. The mushrooms were then broken into small pieces by hand and placed in an electric grinder with blades, where they were ground in short bursts to avoid heating the material. The resulting powder was sifted through a fine sieve to obtain a uniform grain size, and the larger particles were reintroduced into the grinder until the desired fineness was achieved. Dried and powdered plant material was subjected to ethanol extraction (1 gdw/10 mL 70% ethanol) at room temperature for 72 h. After that, the mixture was subjected to ultrasonic treatment for 15 min using a Polonic ultrasonic device (Warsaw, Poland) and filtered through a Whatman filter paper no. 1 (11 µm; Maidstone, UK). Sonication was repeated using an equal volume of ethanol to maximize the extraction efficiency. The combined filtrates were then centrifuged at 10,000 rpm for 15 min. The extracts were subsequently used to evaluate the polyphenolic profile and the antioxidant and antimicrobial activities in vitro [20,21].

### 2.2. Reagents and Standards

The main reagents and standards employed are listed below: potassium sulfate (Supelco Inc., Bellefonte, PA, USA), copper (II) sulfate pentahydrate (Supelco Inc., Bellefonte, PA, USA), sulfuric acid 98%, boric acid (Supelco Inc., Bellefonte, PA, USA), sodium hydroxide (Supelco Inc., Bellefonte, PA, USA), sulfuric acid, 0.1 N volumetric solution (Supelco Inc., Bellefonte, PA, USA), petroleum ether (Chempur, Piekary Śląskie, Poland), standard gallic acid (purity > 98% HPLC) (Sigma-Aldrich, St. Louis, MO, USA), distilled deionized water, 0.1% acetic acid (Honeywell, Raunheim, Germany), acetonitrile, chlorogenic acid, TPTZ (Sigma-Aldrich, St. Louis, MO, USA), ferric chloride, acetate buffer (VWR, Darmstadt, Germany), DPPH (Thermo Scientific, Waltham, MA, USA), fluorescein (Sigma-Aldrich, Saint Louis, MO, USA), copper chloride (Reactivul Bucharest, Bucharest, Romania), ammonium acetate buffer (VWR, Darmstadt, Germany), neocuproine (Nc) (Sigma-Aldrich, Shanghai, China), ABTS (Thermo Scientific, Waltham, WA, USA), potassium persulfate (Thermo Scientific, Waltham, WA, USA), dimethyl sulfoxide (DMSO), crystal violet solution (Sigma-Aldrich, Saint Louis, MO, USA). HPLC-grade acetonitrile and standards (Gallic acid, gentisic acid, protocatechuic acid, p-hydroxybenzoic acid, chlorogenic acid, caffeic acid, p-coumaric acid, and ferulic acid) were purchased from Merck (Darmstadt, Germany), and ultrapure water was purified using the Direct-Q UV system from Millipore (Burlington, MA, USA).

### 2.3. Nutritional and Energy Analysis

To obtain the moisture content, the samples were dried at 105 °C until they reached a constant weight. The ash content was determined by calcination at 600 ± 15 °C for 6 h. The crude protein content of the samples was estimated by the Kjeldahl method. First, the samples were mixed with sulfuric acid 98%, converting the nitrogen in the material into ammonium ions. After digestion, the solution was alkalized and distilled, and the released ammonia was captured in a boric acid solution. The amount of ammonia was then measured by titration with sulfuric acid, 0.1 N, and the percentage of crude protein in the samples was calculated based on the nitrogen content. The crude protein content of the mushrooms was calculated by multiplying the measured nitrogen concentration by a conversion factor of 4.38 [22]. The crude fat content was determined by extracting 3 g of dry mushroom powder with petroleum ether using a Soxhlet apparatus (Gerhardt Soxtherm, Königswinter, Germany). The sample was extracted using the following instrument settings: hot plate temperature 110 °C, boiling time/sample immersion cycle time 30 min, sample rinse cycle time 4 h, solvent evaporation and recycling time 30 min. The extraction cup containing the extract was dried at 103 °C for 1 h in an electric oven (Memmert, Schwabach, Germany), cooled at room temperature in a desiccator, and weighed with an analytical balance (Kern&Sohn GmbH, Balingen, Germany) [23]. The total carbohydrate amount was calculated by difference: total carbohydrates = 100 − (g moisture + g protein + g fat + g ash). The total energy was calculated according to the following equations: energy = 4 × (g protein + g carbohydrate) + 9 (g lipid) [kcal] [24].

### 2.4. Spectrophotometric Analysis

#### 2.4.1. Total Polyphenolic Content (TPC)

The total polyphenol content of the four *C. cornucopioides* extracts was determined using the Folin-Ciocâlteu method. For this procedure, 1 mL of extract or standard gallic acid solution (20, 40, 60, 80, and 100 mg/L) was decanted into a 25 mL volumetric flask containing 9 mL of distilled deionized water. One milliliter of Folin-Ciocâlteu reagent was added to the mixture and stirred. After 5 min, 10 mL of 7% Na_2_CO_3_ solution was added, then the solution was diluted to volume with distilled deionized water and mixed. After incubation for 90 min at room temperature, the absorbance was measured using a UV–Vis spectrophotometer (Agilent Technologies, Cary 60, Santa Clara, CA, USA) against the prepared control (deionized distilled water) at a wavelength of 750 nm. The total polyphenolic content of the mushroom extracts was reported in mg gallic acid equivalent (GAE)/g dw [25,26].

#### 2.4.2. Total Polyphenolcarboxylic Acid Content (TPAC)

The total polyphenolcarboxylic acid content (TPAC) of the *C. cornucopioides* extracts was determined spectrophotometrically using Arnow’s reagent. The assay was performed based on a calibration curve prepared with caffeic acid as a standard (R^2^ = 0.989). Absorbance was measured at 500 nm using a UV–Vis spectrophotometer (Agilent Technologies, Cary 60, Santa Clara, CA, USA). The results were expressed as mg caffeic acid equivalents (CAE)/g dw [25,26].

### 2.5. HPLC-DAD-ESI+ Analysis

Each sample was filtered through a Chromafil Xtra nylon 0.45 µm filter (Macherey-Nagel, Düren, Germany), and 20 μL were injected into the HPLC system. An Agilent 1200 HPLC system (Agilent Technologies, Santa Clara, CA, USA), equipped with a quaternary pump, solvent degasser, autosampler, UV-Vis diode-array detector (DAD), and a 6110 single-quadrupole mass detector, was employed for the analysis. The compounds were separated on a Kinetex XB C18 column, dimensions 4.6 × 150 mm, with 5 μm particles (Phenomenex, Torrance, CA, USA), using mobile phases (A) water + 0.1% acetic acid and (B) acetonitrile + 0.1% acetic acid in the gradient below, for 30 min, at a temperature of 25 °C, with a flow rate of 0.5 mL/min. Gradient (expressed in % B): 0 min, 5% B; 0–2 min, 5% B; 2–18 min, 5–40% B; 18–20 min, 40–90% B; 20–24 min, 90% B; 24–25 min, 90–5% B; 25–30 min, 5% B. The spectral values were recorded in the 200–600 nm range for all peaks. Chromatograms were monitored at 280 nm and 340 nm. Mass spectrometric detection was carried out in positive ESI full-scan mode using the following parameters: capillary voltage of 3000 V, source temperature of 350 °C, nitrogen at 7 L/min, collision energy of 100 V, and a scan range of *m*/*z* 120–1200. Data acquisition and processing were completed using an Agilent ChemStation software (version B.02.01 SR2; Agilent Technologies, Santa Clara, CA, USA) [27,28].

### 2.6. Antioxidant Capacity Assays

The antioxidant activity of *C. cornucopioides* extracts was evaluated using five complementary in vitro methods (FRAP, ABTS, CUPRAC, DPPH, and ORAC), as each test is based on distinct reaction principles and provides a different perspective on the antioxidant capacity of these samples, thus ensuring a comprehensive and comparable characterization of their bioactive potential.

#### 2.6.1. FRAP (Ferric Reducing Antioxidant Power) Assay

The FRAP test is a widely used method for evaluating the total antioxidant capacity of a sample. The principle of the method is based on the reduction of ferric ions (Fe^3+^) to ferric ions (Fe^2+^) under acidic conditions, which subsequently leads to the formation of a stable, blue-colored complex, ferric-tripyridylthiazine (Fe^2+^–TPTZ). This chromogenic reaction produces an intense absorbance measurable at 593 nm. The intensity of the resulting color is directly proportional to the reducing power of the sample. FRAP values are determined by comparing the absorbance variation in the test solution with that of standard solutions containing known concentrations of ferric ions. Initially, the FRAP reagent was prepared using a known volume of TPTZ solution (2.5 mL, 10 mM), ferric chloride solution (2.5 mL, 20 mM), and acetate buffer (25 mL). After its preparation, 180 µL of FRAP reagent was mixed with 20 µL of the tested extract. A blank solution was also created by replacing the sample with distilled water. Absorbance measurements at λmax = 593 nm were performed using a UV-Vis spectrophotometer (Agilent Technologies, Santa Clara, CA, USA) to correlate the color change with antioxidant capacity. A Trolox calibration curve was used to calculate antioxidant activity. The results were displayed in µmoL Trolox equivalents (TE)/g dw [25,26,29].

#### 2.6.2. DPPH Radical Scavenging Activity Assay

The principle of the DPPH method consists of a single electron of the nitrogen atom in DPPH being reduced to the corresponding hydrazine by taking up a hydrogen atom from antioxidants. The DPPH· radical has a remarkably stable and intense color. Due to these properties of the radical, this solution has been used extensively. In order to apply the analysis protocol, 250 µL of 80 µM DPPH solution in methanol was mixed with 35 µL extract in different quantities. The samples were kept in a water bath for half an hour at 40 °C prior to measuring the decrease in absorbance at λmax = 517 nm. The percentage of DPPH radical inhibition was then calculated based on the following formula: % DPPH inhibition = (A_blank_ − A_extract under investigation_/A_blank_) × 100, where A_blank_ represents the absorbance of DPPH radicals + methanol, and A_extract under investigation_ represents the absorbance of DPPH radicals+ tested extract. The results were displayed in µmoL Trolox equivalents (TE)/g dw [25,26,30].

#### 2.6.3. ORAC (Oxygen Radical Absorbance Capacity) Assay

For each sample, 150 µL of freshly prepared fluorescein solution (5 nM) was added to a black flat-bottomed 96-well microplate, followed by 25 µL of extract diluted to different concentrations (ranging from 0 to 200 µg/mL). The plate was incubated for 30 min at 37 °C. After incubation, fluorescence measurements (at 485 nm and 520 nm) were recorded every 100 s for three cycles to determine the baseline signal. Then, 25 µL of 250 mM AAPH (2,2′-azobis(2-amidinopropane) dihydrochloride), a peroxyl radical generator, was added manually using a multichannel pipette. Fluorescence measurements continued for up to 150 min using a FLUOstar Omega multimode plate reader (BMG Labtech, Offenburg, Germany) with Reader Control/MARS Data Analysis software. The antioxidant capacity of the tested extracts was then expressed as Trolox equivalents (TE)/g dw [31].

#### 2.6.4. CUPRAC (Cupric Ion Reducing Antioxidant Capacity) Assay

A 10 mM CuCl_2_ solution was prepared by dissolving 0.085 g of CuCl_2_·2H_2_O in distilled water and bringing the volume to 50 mL. A 1.0 M ammonium acetate (NH_4_Ac) buffer was prepared and adjusted to pH 7.0 by dissolving 19.27 g of NH_4_Ac in water and diluting to 250 mL. The neocuproine (Nc) solution, at a concentration of 7.5 mM, was prepared fresh each day by dissolving 0.039 g of Nc in 96% ethanol and diluting to 25 mL with the same solvent. Trolox, used as a standard antioxidant, was prepared as a 1.0 × 10^−3^ M solution in 96% ethanol. To evaluate the antioxidant activity of the four 1:10 diluted ethanolic extracts, the CUPRAC test was performed as follows: 0.5 mL of Cu(II) solution (1.0 × 10^−2^ M), 0.5 mL of neocuproine (Nc) solution (7.5 × 10^−3^ M), and 0.5 mL of ammonium acetate buffer (1.0 M, pH 7.0) were added to a test tube. Then, 10 μL of each diluted mushroom extract and 0.49 mL of distilled water were added to bring the final volume to 2 mL. The tubes were sealed and incubated at room temperature for 30 min. After the reaction period, the absorbance at 450 nm (A_450_) was measured using a Multiplate reader (Tecan Sunrise, Männedorf, Switzerland), using a control reactant as a reference. The antioxidant capacity of each extract was then calculated and expressed as Trolox equivalents (TE)/g dw, based on the slope of the standard curve [32].

#### 2.6.5. ABTS (2,2′-Azino-Bis(3-Ethylbenzothiazoline-6-Sulfonic Acid)) Assay

The ABTS radical cation (ABTS•^+^) was generated by reacting 5 mL of ABTS stock solution (7 mM) with 5 mL of potassium persulfate 2.45 mM. The mixture was incubated in the dark at room temperature for 16 h to allow complete radical formation. Before use, the resulting solution was diluted with distilled water to obtain an absorbance of 0.700 ± 0.020 at 734 nm and equilibrated at 30 °C. To test the antioxidant capacity, each extract was diluted 1:100 and further diluted with dimethyl sulfoxide (DMSO) to obtain different working concentrations. Then, 5 μL of each diluted extract was mixed with 195 μL of ABTS•^+^ solution in a 96-well plate. The reaction mixture was incubated at room temperature for 6 min, after which the absorbance at 734 nm was recorded using a microplate spectrophotometer (BiotekSynergyHT, Winooski, VT, USA). All measurements were performed in triplicate, with appropriate blank and control reagents included in each assay [33].

### 2.7. Antimicrobial Properties

The antimicrobial properties of *C. cornucopioides* extracts were evaluated in vitro using an agar well diffusion assay and an antibiofilm formation assay. Seven bacterial reference strains were integrated, namely *Staphylococcus aureus* ATCC 25923 (methicillin-susceptible *S. aureus*, MSSA), *Staphylococcus aureus* ATCC 700699 (methicillin-resistant *S. aureus*, MRSA), *Bacillus cereus* ATCC 14579, *Enterococcus faecalis* ATCC 29219, *Escherichia coli* ATCC 25922, *Listeria monocytogenes* ATCC, and *Pseudomonas aeruginosa* ATCC 27853, and one yeast, *Candida albicans.*

### 2.8. Antimicrobial Activity

#### 2.8.1. Agar Well Diffusion Assay Method for Bacteria

The antimicrobial activity of mushroom extracts was evaluated using the agar diffusion method, based on EUCAST methods and the protocols previously described by our team [34,35,36]. Mueller–Hinton agar culture medium was dissolved in distilled water and sterilized by autoclaving at 121 °C for 15 min. After sterilization, the medium was cooled to approximately 50 °C and poured into sterile Petri dishes, ensuring a uniform thickness. A bacterial suspension with a density of 0.5 McFarland was prepared in sterile physiological saline solution and confirmed using an optical densitometer (Biosan, Riga, Latvia), corresponding to a concentration of approximately 10^6^ CFU/mL. Mueller–Hinton (MH) agar plates (Merck, Darmstadt, Germany) were inoculated with the bacterial suspension using a sterile cotton swab, applying the technique in three sectors. Wells with a diameter of 6 mm were made in the agar, and 60 μL of each tested extract was added. In addition, antibiotic disks were placed in the center of the plates: gentamicin (GEN, 10 μg/disk, Oxoid Ltd., Basingstoke, UK). The plates were incubated aerobically for 24 h at 37 °C. After incubation, the inhibition zones were measured in mm using a digital caliper. All tests were performed in triplicate to ensure accuracy and reproducibility.

#### 2.8.2. Agar Well Diffusion Assay Method for Yeast

For this method, 5 agar plates were prepared using the method described above and spread with a uniform layer of *Candida albicans.* Wells were created in the agar using a sterile tool, and the extracts and fluconazole (the reference antifungal) were placed into these wells. The plates were incubated at 30 °C for 48 h. After the incubation, the plates were examined, and the inhibition zone was measured in millimeters using a digital caliper (Mitutoyo, Kawasaki, Japan).

#### 2.8.3. Evaluation of Inhibition Capacity of Bacterial Biofilm Formation

The in vitro antibiofilm potential of *C. cornucopioides* extracts was evaluated against three bacterial strains: *Staphylococcus aureus MSSA*, *MRSA*, and *Escherichia coli*. The evaluation took into account two critical stages of biofilm formation: initial adhesion (T0) and disruption of mature biofilms at 24 h (T24). For the T0 stage (biofilm adhesion), a bacterial suspension with a density of 0.5 McFarland was prepared in sterile physiological saline solution and confirmed using an optical densitometer (Biosan, Riga, Latvia), corresponding to a concentration of approximately 10^6^ CFU/mL, and mixed in equal volumes (100 μL) with the tested extracts in sterile flat-bottomed 96-well microplates. The plates were incubated without shaking for 24 h at 37 °C. In the T24 stage, preformed biofilms were first established by incubating microbial inoculum for 24 h. Subsequently, equal volumes (100 μL) of each extract were added directly to the mature biofilms. After incubation, antibiofilm activity was determined using the crystal violet staining method. The procedure involved removing the supernatant, triple washing with sterile distilled water, drying, fixing with 150 μL of 96% methanol, and staining with 100 μL of 0.1% crystal violet solution. After 20 min at room temperature, the plates were washed again and treated with 150 μL of 100% ethanol to solubilize the dye. The optical density (OD) was measured at 490 nm using a Sunrise™ microplate reader (Tecan Sunrise, Männedorf, Switzerland). The percentage of biofilm inhibition was calculated using the following formula: Inhibition (%) = (OD_control_ − OD_extract_)/OD_control_ × 100. These values (%) allowed the interpretation of the antibiofilm activity in vitro as good (above 50%, ++), weak (0–50%, +), or no inhibition, or enhancement of biofilm development (<0) [37].

### 2.9. Statistical Analysis

Data analysis was performed using GraphPad Prism 8 software(GraphPad Software, LLC San Diego, CA, USA). All determinations were statistically evaluated using one-way ANOVA followed by Tukey’s post hoc test, with a significance threshold set at *p* < 0.05. Pearson correlation coefficients (r) were calculated in Microsoft Excel using the CORREL function. Hierarchical clustering analysis was performed using XLSTAT, applying agglomerative clustering to illustrate regional and composiCORREL (variability among the extracts.

## 3. Results

### 3.1. Nutritional and Energy Analysis

Table 1 contains the results obtained for the analysis of the nutritional composition and energy analysis for the four *C. cornucopioides* samples.

A comparative analysis of *C. cornucopioides* samples collected in Suceava County (CE3 and CE4) revealed notable differences in nutritional composition. The moisture content ranged from 106.3 to 129.0 mg/gdw, with the lowest value recorded in CE3, indicating a higher dry matter content, while the CE4 sample had the highest moisture content. The annual precipitation in the area where the mushrooms were sampled from (CE2 and CE3) was higher in 2023 than in 2022, increasing from approximately 1000–1200 mm to 1200–1400 mm. This suggests a dry climate in 2022, which may directly influence vegetation, soil moisture, and the potential for the accumulation of bioactive compounds in mushrooms harvested in the area. In terms of lipids, the highest concentration was determined in CE3 (41.4 mg/g dw), and the lowest in CE4 (25.8 mg/g dw). Both samples showed a high protein content (>460 mg/g dw), with the maximum recorded in CE3 (473.2 mg/g dw). The carbohydrate content was also higher in both years (≈290 mg/g dw), which was reflected in the energy value, with the CE3 sample recording the highest caloric intake (338.7 kcal/100 g dw) and the CE4 sample showing an intermediate value (324.7 kcal/100 g dw). Overall, the CE3 sample stood out for its optimal nutritional profile, characterized by high protein, lipid, and carbohydrate content, as well as the highest energy value, making it the most promising of the samples analyzed. By contrast, the CE4 sample had the highest moisture content and the lowest lipid concentration but maintained comparable protein and carbohydrate levels. Therefore, samples CE3 and CE1 stood out with a superior nutritional profile, with the highest values of protein, carbohydrates, and energy, while sample CE2 presented intermediate levels. Overall, the statistical analysis showed that sample CE4 had the best results for moisture (*p* < 0.05), fat (*p* < 0.05), and energy (*p* < 0.05), while CE3 had the highest values for protein (*p* < 0.05) and carbohydrates (*p* < 0.05), and CE2 recorded the highest values for ash (*p* < 0.05), with statistically significant differences in all cases, except for CE1 and CE2 for protein and CE1 and CE3 for moisture, where *p* > 0.05.

### 3.2. Spectrophotometric Analysis

The TPC (expressed in mg GAE/g dw) varied significantly between samples depending on pedoclimatic conditions, locations, and years. The highest concentration was observed for the CE3 extract (28.74 ± 0.25 mg GAE/g dw), followed by the CE4 sample (26.64 ± 0.36 mg GAE/g dw), while the CE1 and CE2 samples had a lower content of polyphenols (23.21 ± 0.21 mg GAE/g dw, 23.01 ± 0.02 mg GAE/g dw, respectively). Regarding the concentration of TPAC in the four samples, amounts ranged between 15.96 and 19.74 mg CAE/g dw. The highest values were obtained for CE4 (18.64 mg CAE/g dw), and the CE3 sample (19.74 mg CAE/g dw), and the lowest were measured in extracts of CE1 (15.96 mg CAE/g dw) and CE2 (16.05 mg CAE/g dw) (Table 2). For TPC, the analysis showed that the only comparison without a statistical difference was between CE1 and CE2, where *p* > 0.05. In all other comparisons, the differences were statistically significant, with values of *p* < 0.05. Thus, CE3 showed significantly higher values than CE1 and CE2 (*p* < 0.05), and CE4 also had higher values than CE1 and CE2, also with *p* < 0.05. Direct comparison between CE3 and CE4 indicated a significant difference (*p* < 0.05), with CE3 having the highest values. Tukey post hoc analysis for the TPAC showed that the only comparison without a statistical difference was between CE1 and CE2, where *p* > 0.05, indicating that the values of the two samples were similar. In contrast, all other comparisons showed statistically significant differences, with *p* < 0.05.

### 3.3. HPLC-DAD-ESI+ Analysis

The results regarding the profile of phenolic compounds by HPLC analysis of extracts from *C. cornucopioides* are shown in Table 3. The retention time, UV-VIS spectra, and main molecular ion were used to identify and quantify the phenolic compounds.

Thus, eight compounds belonging to two different subclasses were identified. The hydroxybenzoic acid subclass included gallic acid, gentisic acid, protocatechuic acid, and p-hydroxybenzoic acid, while the hydroxycinnamic acid subclass comprised chlorogenic acid, caffeic acid, ferulic acid, and *p*-coumaric acid. In sample CE1, the highest content of polyphenolic acids was observed for gentisic acid (7575.90 μg/g dw), followed by protocatechuic acid (1943.02 μg/g dw), whereas the lowest concentration was recorded for ferulic acid (295.45 μg/g dw). Sample CE2 also showed a high content of gentisic acid (6597.43 μg/g dw), followed by p-hydroxybenzoic acid (3244.10 μg/g dw) and gallic acid (206.16 μg/g dw). Samples CE3 and CE4 were characterized by gentisic acid as the predominant component (8429.46 μg/g dw and 8057.70 μg/g dw, respectively), followed by protocatechuic acid (3775.04 μg/g dw and 4393.65 μg/g dw, respectively). In CE3, the lowest concentration was gallic acid (518.44 μg/g dw), whereas in CE4, ferulic acid was present at the lowest level (375.15 μg/g dw). Notably, all pairwise comparisons between the CE1–CE4 extracts showed statistically significant differences (*p* < 0.05) across all evaluated phenolic compounds.

### 3.4. Antioxidant Capacity

Given the confirmed nutritional relevance of the tested samples, highlighted by their high content of bioactive compounds, the present study aimed to further investigate their antioxidant potential. Thus, the results obtained from the evaluation of the antioxidant activity of the samples of *C. cornucopioides,* using the DPPH, FRAP, CUPRAC, ORAC, and ABTS methods, are presented in Table 4.

It can be observed that the extract from CE3 has the highest antioxidant potential of all extracts tested by the DPPH (23.03 ± 1.34 μM TE/g dw), CUPRAC (258.31 ± 12.89 μM TE/g dw), ORAC (147.06 ± 2.92 μM TE/g dw), and ABTS (48.05 ± 1.26 μM TE/g dw) methods, with FRAP ranking third in terms of efficiency (24.47 ± 8.28 µM TE/g dw). The extract with the lowest antioxidant efficiency was identified as being that from CE2, where the following values were obtained: 8.31 ± 2.21 μM TE/g dw (DPPH), 83.13 ± 9.45 μM TE/g dw (CUPRAC), 123.96 ± 2.93 μM TE/g dw (ORAC), and 32.28 ± 3.14 μM TE/g dw (ABTS). Using the FRAP method, this extract ranked second in terms of efficacy (29.83 ± 1.98 µM TE/g dw), ahead of the extract from CE3. According to statistical analysis, of the four extracts, CE3 consistently showed the best antioxidant values in most tests (DPPH, FRAP, CUPRAC, ORAC, ABTS), with statistically significant differences compared to CE1, CE2, and CE4 (*p* < 0.05), while CE1 and CE4 frequently showed similar values, with no significant differences between them (*p* > 0.05) in the DPPH, ORAC, or ABTS tests, and in the FRAP test, most pairs showed no significant differences (*p* > 0.05), confirming that extract CE3 was overall the most potent, followed by CE4, while CE1 and CE2 were generally inferior in terms of antioxidant activity.

Pearson correlation analysis revealed a strong association between total polyphenol content and antioxidant activity, as determined by DPPH, FRAP, ABTS, and ORAC (r = 0.83–0.95), suggesting that polyphenols were the main contributors to the antioxidant capacity of the extracts. In contrast, the CUPRAC method showed a strong but negative correlation (r = −0.80), indicating a different sensitivity of this method to the profile of phenolic compounds in the extracts.

**Figure 1:**
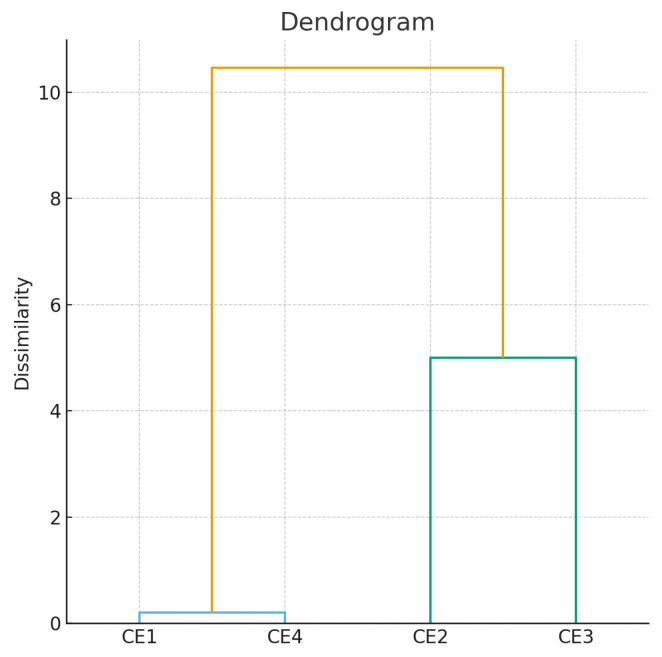
The dendrogram of the hierarchical classification of extracts CE1–CE4 according to antioxidant activity. Blue line—extremely similar; Yellow line—different; Green line—moderately similar.

Hierarchical clustering analysis separated the extracts into two distinct groups: CE1, CE3, and CE4 formed a cluster with high levels of phenolic compounds and antioxidant activity, while CE2 was separated into an individual cluster, reflecting a significantly different antioxidant profile (Figure 1).

### 3.5. Antimicrobial Activity

Regarding the in vitro antimicrobial activity of the analyzed CE extracts against the reference strains, the results obtained by the agar well diffusion method and broth microdilution assay are displayed in Table 5 and Table 6, respectively.

The CE1-4 extracts displayed in vitro antimicrobial potential towards all tested organisms except for *Pseudomonas aeruginosa* (Table 5). Furthermore, this biological potential was significantly lower (*p* < 0.01) compared to the positive controls (gentamicin and fluconazole). Still, the CE1–4 extracts displayed in vitro inhibitory activity against Gram-positive bacterial growth. The highest values obtained for the diameter of the inhibition zone (DIZ) were noticed against *Bacillus cereus* (ranging from 16.00 to 17.00 mm) and MSSA (ranging from 15.00 to 16.50 mm). Against MRSA, the inhibition zones were slightly smaller (14.25–15.25 mm), but the extracts still showed significantly higher activity compared to the Gram-negative strain, *E. coli* (*p* < 0.05). No significant differences were observed when comparing the CE1-4 extracts’ inhibitory potential against MRSA vs. MSSA, MRSA vs. *B. cereus*, or MSSA vs. *B. cereus* (*p* > 0.05). Interestingly, there were no significant differences (*p* > 0.05) between the CE1-4 extracts’ activity (CE1 vs. CE2, CE1 vs. CE3, CE1 vs. CE 4, CE2 vs. CE 3, CE2 vs. CE 4, CE3 vs. CE 4) against MSSA, MRSA, or *B. cereus.* In contrast, a significantly higher inhibitory effect was recorded for CE4 (Suceava 2023) when tested against *E. faecalis* compared to the other CE extracts and also to the positive control, gentamicin (*p* < 0.05).

Based on the obtained results, the CE extracts demonstrated the most intense inhibitory activity against the Gram-positive bacteria, namely *B. cereus*, MSSA, and MRSA, while their antibacterial potential against the Gram-negative strains was found to be relatively low (DIZ ranging from 9.00 ± 0.00 to 10.00 ± 0.00 mm for *Escherichia coli*) or absent (DIZ = 0 mm for *Pseudomonas aeruginosa*). These findings suggest that the extracts may contain compounds with potential against *Staphylococcus aureus* and *Bacillus cereus*.

An in vitro inhibitory effect was also recorded against *Candida albicans,* with all four CE extracts exhibiting inhibition zones of 10.00 ± 0.00 mm. These results indicate that the tested CE extracts may display anti-*C. albicans* activity, but the values of DIZ were still significantly lower compared to the positive control, fluconazole (21.00 ± 0.00 mm) (*p* < 0.01).

The CE extracts were further evaluated by employing the broth microdilution method, with Table 6 presenting the minimum inhibitory (MIC) and bactericidal (MBC) concentrations against the tested microorganisms. The determined MIC, MBC, and MFC values corresponded to the highest tested concentrations for *Enterococcus faecalis*, *Listeria monocytogenes, Escherichia coli,* and *Candida albicans,* while the third or second-highest concentrations were recorded for MSSA, MRSA, and *Bacillus cereus*.

The results of the bacterial biofilm formation inhibition capacity of the analyzed extracts on the reference bacteria are shown in Table 7.

The antibiofilm activity of CE extracts was investigated against MSSA, MRSA, and *Escherichia coli* (Table 7), employing a previously published method. This assay provides values for the biofilm inhibition percentages (IP) that are indicative of a qualitative characterization of the biological effect as good (above 50%, ++), poor (0–50%, +), or no inhibition, or even as promoting biofilm formation (<0) [38]. All CE extracts only displayed a good inhibition against biofilm formation (++)against the MSSA reference strain and only at the T24 stage, corresponding to the disruption of pre-formed 24 h biofilms. The inhibitory effect against MRSA and *E. coli* was poor or absent (Table 7).

## 4. Discussion

*C. cornucopioides* is an edible wild mushroom, poorly known in Europe as a functional food. This is because its chemical composition and bioactivities are poorly known, and therefore, the possibility of using it in medicine is limited. In recent years, several works have been directed towards the discovery of the composition and functional properties of black trumpet, but the results remain scarce and poor, and have significant differences. In this context, the present study aims to deepen the knowledge of the nutritional value of this species, providing a novel further argument for considering it an important food source and medicine.

There are several nutritional studies on this mushroom species that have assessed the influence of processing on its nutritional properties. Thus, the nutritional profile of *C. cornucopioides* has revealed a balanced composition of macronutrients, with carbohydrates as the predominant metabolites, followed by proteins, ash, and lipids [10,11,12]. Thermal processing markedly influences these parameters, inducing structural and chemical modifications that affect nutrient stability and bioavailability. Cooking methods such as steaming, boiling, microwaving, and frying have been shown to alter its protein, lipid, and mineral contents. Protein levels decreased significantly across all treatments (*p* < 0.05), ranging from 9.98 g/100 g dw after microwaving to 16.02 g/100 g dw following frying, due mainly to denaturation, hydrolysis, and the leaching of nitrogen compounds. Lipid content declined during steaming and boiling but increased after frying due to oil absorption, while ash content was reduced, most notably after frying, likely because of mineral losses during soaking and cooking [12,39,40,41].

The reported caloric value of *C. cornucopioides* ranges between 248.0 and 413.2 kcal/100 g, reflecting its moderate energy density linked to its high moisture (62.5–93.2%) and low fat levels (4.9–6.9% dw) [9,42,43,44,45,46]. Its protein content exhibits wide variation (11.8–69.4% dw), largely dependent on the nitrogen-to-protein conversion factor applied in Kjeldahl analysis (4.38 vs. 6.25) [9,43,44,47]. Notably, *C. cornucopioides* displayed the highest total soluble protein (126.6 mg/g) among 15 edible mushrooms tested by Turfan et al. (2018) [40]. Its carbohydrate levels range from 6.2% to 45.6% dry matter, influenced by protein and lipid fluctuations, and include glucose, fructose, sucrose, mannitol, and β-glucans—important dietary fibers with recognized health benefits [33,35,40]. The ash fraction (10.1–17.4%) reflects significant mineral diversity, correlated with soil composition and environmental factors [48].

The results obtained in the present study for the determination of TPC are higher than those published by other authors (11.234 mg/g dry extract) [15], but lower than those found in mushrooms from Serbia (4.39 g gallic acid/100 g dry ethanolic extract) [16]. TPAC represents the most frequently found phenolic compounds in mushrooms, and are associated with numerous bioactive properties (immunomodulatory, antioxidant, hypoglycemic, hypocholesterolemic, detoxifying, antitumor, etc.), and thus, an increase in interest has been observed in the use of some mushrooms as potential nutraceuticals, but also as functional foods. From the literature we consulted, no data were found regarding the TPAC in the composition of *C. cornucopioides*. A comparison of TPC revealed that samples CE3 and CE4 presented significantly higher amounts of active phenolic metabolites, suggesting the existence of favorable conditions for the biosynthesis and accumulation of polyphenols with high antioxidant potential. Consequently, this mushroom species can be considered a valuable source of polyphenolic compounds, including caffeic acid derivatives, which significantly contribute to its bioactive profile and nutraceutical potential.

A previously performed HPLC analysis revealed the presence of homogentisic acid in the composition of *C. cornucopioides* in low amounts, along with minor levels of gallic acid, protocatechuic acid, and p-hydroxybenzoic acid, as well as other phenolic acids such as chlorogenic, caffeic, and ferulic acids [14]. Notably, gentisic acid had not previously been reported in this species, whereas in our study, it was identified as a major component. A more recent study focusing solely on the phenolic profile of *C. cornucopioides* extract revealed the presence of several individual phenolic acids in varying concentrations: gallic acid and p-coumaric acid (both 3.5 mg/g), chlorogenic acid (2.7 mg/g), syringic acid (0.8 mg/g), and ferulic acid (4.7 mg/g). Caffeic acid was not quantified in this extract [4], whereas in our study, its content ranged from 15.96 ± 0.14 mg CAE/g dw to 19.74 ± 0.26 mg CAE/g dw.

The results obtained for antioxidant activity tests differ depending on the testing method [48]. A Romanian study evaluating the antioxidant effects of an 80% ethanolic extract from *C. cornucopioides* was conducted using four standardized methods, with results expressed in mg of ascorbic acid equivalent (mg AAE/g) or Na_2_-EDTA (mg Na_2_-EDTA/g), reported per gram of dry extract. The free radical scavenging activity was tested using the DPPH and ABTS methods and obtained values of 18.11 ± 0.74 mg AAE/g and 295.34 ± 0.49 mg AAE/g, respectively. The reducing capacity of oxidizing compounds was evaluated using the modified FRAP test, and the chelating activity of metal ions was determined using the ferric ion chelation test. These results indicate a significant antioxidant potential of the ethanolic extract, with high activity in free radical scavenging tests (DPPH and ABTS) and moderate capacity for reducing metal ions (modified FRAP) and chelating them, suggesting promising value in functional or therapeutic applications [15]. The antioxidant potential of the *C. cornucopioides* extract was evaluated by three other tests: DPPH, superoxide anion scavenging, and FRAP. The IC_50_ value for DPPH was 19.7 ± 1.1 µg/mL, while for superoxide anion radicals, the IC_50_ reached 221.8 ± 3.1 µg/mL, indicating lower efficacy in neutralizing superoxide radicals compared to DPPH. The reducing power of the extract, measured as absorbance at 700 nm, was concentration-dependent, although relatively low, with values of 0.1 ± 0.0 even at the highest concentration tested (2000 µg/mL). Compared to the positive control (ascorbic acid), which demonstrated significantly higher antioxidant performance in all tests, the extract showed a statistically significant difference (*p* < 0.05), confirming moderate but relevant antioxidant activity. These results suggest that the extract from *C. cornucopioides* is less effective than ascorbic acid under the tested conditions [4]. Another study analyzing the antioxidant effects of 12 mushrooms, including *C. cornucopioides*, showed moderate antioxidant capacity. The CUPRAC value was 10.569 ± 0.019 mg TE/mg dry weight (compared to our values), indicating an acceptable capacity to reduce copper ions, while FRAP reached values of 11.958 ± 0.072 mmol Fe^2+^/mg dry weight. The total polyphenol content reflects a significant presence of phenolic compounds. These results suggest that the mushroom *C. cornucopioides* possesses relevant antioxidant properties, comparable to other commonly consumed edible mushrooms [16]. To date, the antioxidant capacity of *C. cornucopioides* has not been evaluated using the ORAC method. *C. cornucopioides* extracts showed exceptionally high antioxidant capacities as measured by the ORAC test. These levels are higher than those observed in well-known medicinal mushrooms such as Chaga (0.63 ± 0.12 μmol TE/mg) and Maitake (0.19 ± 0.08 μmol TE/mg). Even Chaga, the species with the highest ORAC in the comparative group, demonstrated a much lower antioxidant potential than *C. cornucopioides* when expressed per unit volume [18]. Notably, the CE3 extract showed the highest ORAC activity, indicating possible regional or ecological influences on antioxidant content. This high ORAC response positions *C. cornucopioides* as a promising source of potent natural antioxidants, potentially surpassing some of the most studied medicinal mushrooms in mitigating oxidative stress.

With regard to antimicrobial activity, our results pointing out the *in vitro* efficacy of *C. cornucopioides* against certain Gram-positive bacteria are in agreement with data reported in the scientific literature. A study performed in Serbia in 2019 demonstrated that a *C. cornucopioides* extract proved to be active against reference strains such as *Staphylococcus aureus* ATCC 25923, *B. subtilis* ATCC 6633, *B. cereus* ATCC 10987, *Escherichia coli* ATCC 25922, *Proteus mirabilis* ATCC 12453, *Aspergillus niger* ATCC 16888, *Candida albicans* ATCC 10259, *Penicillium italicum* ATCC 10454, *Mucor mucedo* ATCC 20094, and *Trichoderma viride* ATCC 13233. The minimum inhibitory concentrations (MICs) ranged from 0.1 to 0.2 mg/mL for bacteria and 5–10 mg/mL for molds. Among the microorganisms examined, *Bacillus cereus* and *Bacillus subtilis* proved to be the most sensitive, with a MIC of 0.1 mg/mL. The antimicrobial efficacy of *C. cornucopioides* was influenced by both the concentration of the extract and the specific microorganisms tested. In these tests, inhibitory effects were observed against both Gram-positive and Gram-negative bacteria, although Gram-negative strains showed greater resistance. In general, Gram-negative bacteria are usually more resistant than Gram-positive bacteria. Compared to bacteria, fungi showed lower susceptibility, which can be explained by the more complex composition of their cell walls.

A comprehensive study conducted in Tanzania investigated the antibacterial properties of crude ethanol extracts derived from eight wild edible mushrooms against six microorganisms. Based on the diameter of inhibition zones (DIZ, mm) obtained by the agar disk diffusion method, five mushrooms (*Cantharellus luteopunctatus*, *Cantharellus* sp., *Craterellus* sp., *Lactarius kabansus,* and *Afrocantharellus platyphyllus*) showed notable antimicrobial activity, while *Amanita* sp., *Lentinus* sp., and *Termitomyces* sp. showed no inhibitory effect against any of the microorganisms tested. *Craterellus* sp. demonstrated the strongest antibacterial activity, with the largest inhibition zone recorded at 17.00 ± 0.57 mm against *S. aureus*, followed by 14.33 ± 0.33 mm against *S. typhi* and 13.00 ± 0.57 mm against *E. coli*. Its activity against *P. aeruginosa* and *C. albicans* was also significant, at 12.33 ± 0.33 mm and 12.67 ± 0.33 mm, respectively. In comparison, *C. luteopunctatus* produced inhibition zones of 13.33 ± 0.33 mm (*S. aureus*), 12.33 ± 0.33 mm (*S. typhi*), and 15.33 ± 0.33 mm (*C. albicans*), the latter exceeding the positive antifungal control (Nystatin, 14.67 ± 0.33 mm). *A. platyphyllus* showed the lowest antimicrobial effect, with a minimum inhibition of 9.70 ± 0.33 mm against *E. coli. Bacillus subtilis* was resistant to all ethanolic extracts. The MIC was also tested in this study, where *Craterellus* sp. extracts consistently showed moderate inhibitory activity, especially against *Staphylococcus aureus*, with the lowest MIC value of 8.3 ± 2.0 mg/mL, indicating superior antibacterial efficacy compared to other fungi. For *Escherichia coli*, *Craterellus* sp. showed a MIC of 33.3 ± 8.3 mg/mL, similar to that of *Cantharellus* sp., but higher than that of *C. luteopunctatus* and *A. platyphyllus*. Against *Salmonella typhi*, *Craterellus* sp. had a MIC of 20.8 ± 4.2 mg/mL, slightly higher than *Cantharellus* sp. (5.2 ± 1.1 mg/mL), but comparable to *C. luteopunctatus* and *L. kabansus.* The extract also demonstrated activity against *Pseudomonas aeruginosa*, with a MIC of 83.3 ± 16.6 mg/mL, reflecting moderate susceptibility. For the yeast *Candida albicans*, *Craterellus* sp. exhibited a MIC of 33.3 ± 8.3 mg/mL, showing notable antifungal potential, although slightly weaker than *Cantharellus luteopunctatus* (10.4 ± 2.1 mg/mL) [19]. A 2018 study investigated the antimicrobial activity of *C. cornucopioides* collected from Trabzon, Turkey, using both aqueous and methanolic extracts. The inhibitory effect was measured as the zone of inhibition (mm) determined by the agar disk diffusion method. The aqueous extract of *C. cornucopioides* showed minimal activity, with values of 6 mm against all bacteria tested (*E. coli*, *S. aureus*, *B. subtilis*, *B. licheniformis*, *A. tumefaciens*, and *E. faecalis*). In contrast, the methanolic extract demonstrated slightly higher antimicrobial activity, producing values between 7 and 8 mm, with the highest bacterial inhibition observed against *B. subtilis* (8 mm). For comparison, gentamicin produced considerably larger inhibition zones, ranging from 23 to 27 mm in all tested strains [17].

While the level of the antimicrobial efficacy varies when comparing the testing protocols and the extraction method, *C. cornucopioides* is known for its content of compounds such as ferulic acid, gallic acid, and p-coumaric acid, substances recognized for their potent antimicrobial effects against a wide range of bacteria and fungi [4]. Limitations of the present study represent future research directions and are related to in vivo testing of the biological activities of these extracts, together with the evaluation of their toxicological profile.

## 5. Conclusions

The analysis of the mushroom *C. cornucopioides*-derived extracts in this study revealed a valuable nutritional profile, characterized by a high content of protein (473.2 ± 0.8 mg/gdw) and carbohydrates (297.1 ± 0.3 mg/gdw) and a relatively low level of lipids, underlining its potential as a healthy food source. Spectrophotometric quantification of total polyphenols and caffeic acid derivatives, combined with the identification and quantification of individual phenolic compounds by HPLC-DAD-ESI^+^, demonstrated a biologically relevant phytochemical composition. CE3 stands out as the extract with the best antioxidant properties and highest phenolic content. CE3 showed the highest values for most antioxidant methods (DPPH, FRAP, CUPRAC, ORAC, ABTS) and a higher TPC level, and the strongly positive correlations (r > 0.80–0.95) between TPC and antioxidant activity support this superiority. In addition, the CE1-4 extracts expressed in vitro antimicrobial potential towards all tested organisms except for *Pseudomonas aeruginosa.* Taken together, these findings underscore the nutritional, antioxidant, and antimicrobial potential of *C. cornucopioides*, supporting its classification as an edible but under-explored mushroom species with promising applications in both the food and pharmaceutical industries.

## Figures and Tables

**Table 1:** Nutritional composition and energy value of samples of *C. cornucopioides*.

Samples	Moisture (mg/g dw)	Total Fat (mg/g dw)	Crude Protein (mg/g dw)	Ash (mg/g dw)	Carbohydrates (mg/g dw)	Energy (kcal/100 g dw)
CE1	103.1 ± 0.2	34.6 ± 0.1	469.7 ± 1.2	95.5 ± 0.1	297.1 ± 0.3	337.86 ± 0.02
CE2	125.3 ± 0.3	39.4 ± 0.2	467.1 ± 0.7	104.0 ± 0.2	264.2 ± 0.4	327.98 ± 0.04
CE3	106.3 ± 1.5	41.4 ± 0.1	473.2 ± 0.8	98.7 ± 0.1	280.4 ± 1.0	338.7 ± 0.09
CE4	129.0 ± 0.2	25.8 ± 0.7	462.1 ± 0.5	91.5 ± 0.2	291.6 ± 0.4	324.7 ± 0.03

Note: Each value is the mean ± SD of three independent measurements, *p* < 0.05 (except CE1 vs. CE2 for protein and CE1 vs. CE3 for moisture).

**Table 2:** Total polyphenolic and caffeic acid derivatives contents in *C. cornucopioides* extracts.

Sample	TPC (mg GAE/g dw)	TPAC (mg CAE/g dw)
CE1	23.21 ± 0.21	15.96 ± 0.14
CE2	23.01 ± 0.02	16.05 ± 0.45
CE3	28.74 ± 0.25	19.74 ± 0.26
CE4	26.64 ± 0.36	18.64 ± 0.26

Note: Each value is the mean ± SD of three independent measurements, *p* < 0.05 (except CE1 vs. CE2).

**Table 3:** Phenolic compounds identified in the four extracts of *C.cornucopioides* by HPLC analysis.

No.	R_t_ (min)	UV λ_max_ (nm)	[M+H]^+^ (m/z)	Compound	CE1 μg/g dw	CE2 μg/g dw	CE3 μg/g dw	CE4 μg/g dw
1	4.63	275	171	Gallic acid	670.12 ± 0.26	206.16 ± 0.66	518.44 ± 0.63	1467.17 ± 0.29
2	8.39	280	155	Gentisic acid	7575.9 ± 0.61	6597.43 ± 0.47	8429.46 ± 1.02	8057.70 ± 0.69
3	9.17	280	155	Protocatechuic acid	1943.02 ± 0.41	759.34 ± 0.52	3775.04 ± 0.51	4393.65 ± 0.58
4	11.66	280	139	p-Hydroxybenzoic acid	1327.39 ± 0.35	3424.10 ± 0.65	3254.58 ± 0.91	1110.28 ± 0.44
5	12.33	330	355	Chlorogenic acid	392.86 ± 0.67	569.97 ± 0.99	738.22 ± 0.43	446.00 ± 0.33
6	13.83	330	181	Caffeic acid	384.01 ± 0.98	1003.89 ± 0.09	760.36 ± 0.63	410.57 ± 0.22
7	15.50	310	165	p-Coumaric acid	415.00 ± 0.59	658.53 ± 0.11	600.97 ± 0.69	388.43 ± 0.99
8	17.31	331	195	Ferulic acid	295.45 ± 0.73	707.23 ± 0.29	671.81 ± 0.77	375.15 ± 0.26

Note: Values represent the mean ± standard deviations of three independent measurements, R_t_—retention time.

**Table 4:** Antioxidant activity of extracts from *C.cornucopioides* mushrooms.

Sample	DPPH (μM TE/g dw)	FRAP (µM TE/g dw)	CUPRAC (μM TE/g dw)	ORAC (μM TE/g dw)	ABTS (μM TE/g dw)
**CE1**	13.01 ± 2.08	30.60 ± 0.25	157.61 ± 32.02	133.75 ± 0.49	34.78 ± 2.20
**CE2**	8.31 ± 2.21	29.83 ± 1.98	83.13 ± 9.45	123.96 ± 2.93	32.28 ± 3.14
**CE3**	23.03 ± 1.34	24.47 ± 8.28	258.31 ± 12.89	147.06 ± 2.92	48.05 ± 1.26
**CE4**	12.98 ± 0.61	20.97 ± 3.38	185.62 ± 13.42	132.77 ± 2.72	38.68 ± 0.63

Notes: Each value is the mean ± SD of three independent measurements; TE: Trolox equivalents.

**Table 5:** In vitro antimicrobial activity of CE extracts investigated by agar well diffusion method.

Extracts	Diameters of Inhibition Zone (mm)							
	MSSA	MRSA	*Bacillus cereus*	*Enterococcus faecalis*	*Listeria monocytogenes*	*Escherichia coli*	*Pseudomonas aeruginosa*	*Candida* *albicans*
**CE1**	15.75 ± 0.43 ^a^	15.00 ± 0.00	16.00 ± 0.00 ^b^	11.75 ± 0.4 3	11.00 ± 0.0 ^a^	10.00 ± 0.00 ^a^	0	10.00 ± 0.00 ^a^
**CE2**	15.00 ± 0.00 ^a^	14.25 ± 0.43 ^b^	16.75 ± 0.83 ^b^	9.00 ± 0.00	14.25 ± 0.43 ^a^	9.00 ± 0.00 ^a^	0	10.00 ± 0.00 ^a^
**CE3**	16.50 ± 0.50 ^b^	15.25 ± 0.43	17.00 ± 0.71	10.75 ± 0.43	10.00 ± 0.00 ^a^	9.00 ± 0.00 ^a^	0	10.00 ± 0.00 ^a^
**CE4**	15.00 ± 1.00 ^a^	14.25 ± 0.43 ^b^	17.00 ± 00	16.50 ± 0.50 ^b^	14.00 ± 1.00 ^a^	9.00 ± 0.00 ^a^	0	10.00 ± 0.00 ^a^
**Gentamicin**	19.00 ± 0.00	17.00 ± 0.25	20.00 ± 0.00	10.00 ± 0.00	22.00 ± 0.50	19.00 ± 0.00	18.00 ± 0.00	nt
**Fluconazole**	nt	nt	nt	nt	nt	nt	nt	21.00 ± 0.00

Note: The values of the diameter of the inhibition zone are presented as means of duplicate determinations (*n* = 2) ± standard deviations. MSSA—methicillin-susceptible *Staphylococcus aureus*, and MRSA—methicillin-resistant *Staphylococcus aureus*. Lowercase letters in the same column point out significant differences: ^a^ *p* < 0.01 (extract vs. gentamicin); ^b^ *p* < 0.05 (extract vs. fluconazole);. Gentamicin (10 µg/disk) and fluconazole (25 µg/disk) were included as positive controls; nt: not tested.

**Table 6:** In vitro antimicrobial activity of CE extracts investigated by broth microdilution method.

	MSSA		MRSA		*Bacillus* *Cereus*		*Enterococcus* *Faecalis*		*Listeria* *Monocytogenes*		*Escherichia* *Coli*		*Candida* *Albicans*	
	MIC	MBC	MIC	MBC	MIC	MBC	MIC	MBC	MIC	MBC	MIC	MBC	MIC	MFC
**CE1**	0.23	0.46	0.46	0.46	0.46	0.46	0.93	0.93	0.93	0.93	0.93	>0.93	0.93	>0.93
**CE2**	0.23	0.23	0.47	0.47	0.47	0.47	0.94	0.94	0.47	0.94	0.94	>0.94	0.94	>0.94
**CE3**	0.29	0.29	0.58	0.58	0.58	0.58	1.16	1.16	1.16	1.16	1.16	>1.16	1.16	>1.16
**CE4**	0.27	0.27	0.27	0.27	0.54	0.54	0.27	0.54	0.54	1.09	1.09	>1.09	1.09	>1.09
**Gentamicin** **MIC (mg/L)**	3		4		3		3		3		4		nt	
**Fluconazole** **MIC (mg/L)**	nt		nt		nt		nt		nt		nt		8	

Note: MIC: minimum inhibitory concentration (µmol GAE/mL)/; MBC: minimum bactericidal concentration (µmol GAE/mL); MFC: minimum fungicidal concentration (µmol GAE/mL); nt: not tested.

**Table 7:** Anti-biofilm antimicrobial activity of *C. cornucopioides* extracts against three microbial strains (T0 = 24 h incubation time, T24 = 48 h incubation time, − inactive; + weak, and ++ good antibiofilm activity).

	Inhibition Percentage (%)					
Sample	MSSA		MRSA		*Escherichia coli*	
	T0	T24	T0	T24	T0	T24
CE1	+	++	−	+	−	−
CE2	+	++	−	−	−	+
CE3	++	++	+	+	−	+
CE4	+	++	−	−	−	−
Gentamicin	+	++	+	++	−	++

Note: MSSA—methicillin-susceptible *Staphylococcus aureus*, and MRSA—methicillin-resistant *Staphylococcus aureus*.

## Data Availability

The original contributions presented in the study are included in the article; further inquiries can be directed to the corresponding author.
